# Expression of estrogen receptors and androgen receptor and their clinical significance in gastric cancer

**DOI:** 10.18632/oncotarget.16582

**Published:** 2017-03-28

**Authors:** Wenbo Tang, Rujiao Liu, Yan Yan, Xiaoli Pan, Minjun Wang, Xiaotian Han, Hui Ren, Zhe Zhang

**Affiliations:** ^1^ Department of Medical Oncology, Fudan University Shanghai Cancer Center, Shanghai, P.R. China; ^2^ Department of Oncology, Shanghai Medical College, Fudan University, Shanghai, P.R. China; ^3^ Department of Neurology, Zhongshan Hospital, Fudan University, Shanghai, P.R. China; ^4^ Shanghai Key Laboratory of Bioactive Small Molecules, Department of Pharmacology, School of Pharmacy, Fudan University, Shanghai, P.R. China; ^5^ Department of Gynecologic Oncology, Fudan University Shanghai Cancer Center, Shanghai, P.R. China; ^6^ Department of Breast Surgery, Lanzhou General Hospital, Lanzhou, P.R. China

**Keywords:** gastric cancer, estrogen receptor, androgen receptor, epithelial-mesenchymal transition

## Abstract

Despite the mounting studies exploring the role of estrogen receptor alpha (ERα), estrogen receptor beta (ERβ) and androgen receptor (AR) in gastric cancer (GC), there remain controversies in those findings. The present study investigated the expression of ERα, ERβ and AR in Chinese gastric cancer by immunohistochemistry, analyzed their clinical relevance in gastric cancer, and examined the potential mechanisms by which ERα and AR modulated GC progression. The positive rate of ERα, ERβ and AR in GC tissues was 6% (9/150), 93.5% (143/153), and 42.4% (59/139), respectively. The expression of ERα was an independent unfavorable risk factor for overall survival (OS) (hazard ratio [HR] = 3.639, 95% confidence interval [CI] = 1.432-9.246, *p* = 0.007) for GC patients. Moreover, AR was borderline significantly associated with poor progress free survival (PFS) after adjustment with other variables (HR = 1.573, 95% CI = 0.955-2.592, *p* = 0.075). Knockdown of ERα inhibited the proliferation, migration and invasion of GC cells possibly via modulating the expression of p53, p21, p27, cyclin D1 and E-cadherin. Downregulation of AR suppressed the migration and invasion of GC cells and inhibited the epithelial-mesenchymal transition (EMT) associated pathways.

**Conclusion:**

The present study showed that positive ERα was associated with poor prognosis of Chinese GC patients. ERα might modulate the proliferation, migration and invasion via regulating the expression of p53, p21, p27, cyclin D1 and E-cadherin. ERα could be a valuable prognostic biomarker and promising therapeutic target for Chinese GC patients.

## INTRODUCTION

Gastric cancer (GC) is the fifth most common cancer and the third leading cause of cancer-related death in the world [1]. A large number of patients are diagnosed with GC at advanced stage with poor prognosis. Even though progress has been achieved in recent years in the treatment of GC, the benefit is still modest. For example, trastuzumab combined with chemotherapy markedly improved the survival of patients with human epidermal growth factor receptor 2 (HER2)-positive metastatic GC, the median overall survival was only 13.8 months [2]. All these concerns illustrate an urgent need for novel therapies and biomarkers to help identify GC patients who will benefit from specific treatments.

In the past decades, hormonal therapy has been well established in the treatment of hormone-dependent tumors such as prostate cancer and breast cancer [3, 4]. However, the role of hormone receptors in tumors located innon-target organs, including GC, remains largely unknown.

The relationship between hormone receptors and GC was first reported by Tokunaga et al [5]. Since then, mounting studies have investigated the expression of sex hormone receptors including estrogen receptor alpha (ERα), estrogen receptor beta (ERβ) and androgen receptor (AR), as well as their prognostic implications in GC [6–20]. However, the results remain inconclusive and controversial. For instance, some studies claimed that ERα was not expressed while ERβ was expressed abundantly in GC [10], whereas others showed that both receptors were expressed [7, 9, 11, 17, 18]. In addition, some studies indicated ER expression was associated with advanced stage and poor survival whereas others drew totally different conclusion [7, 14, 17, 18]. Besides, there were limited researches on the role of AR in GC [18–20]. The present study investigated the prognostic role and potential mechanisms of ERα, ERβ and AR in GC patients of Chinese population, aiming to provide evidence justifying the possibility of ERα as a novel therapeutic target.

## RESULTS

### ERα, ERβ and AR expression in GC tissues

**Figure 1:**
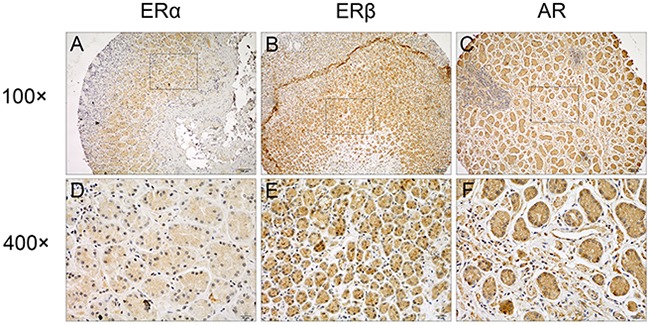
Representative immunostaining of ERα, ERβ and AR in GC tissues Positive staining of **(A, D)** ERα, **(B, E)** ERβ, and **(C, F)** AR in GC tumors were shown. Original magnification, 100×for (A), (B) and (C), 400×for (D), (E) and (F).

The positive rate of ERα, ERβ and AR in GC tissues was 6.0% (9/150), 93.5% (143/153), and 42.4% (59/139), respectively (Table 1). Representative staining results of ERα, ERβ and AR were presented in Figure 1, indicating the expression of the three receptors in both cytoplasm and nucleus. The correlations of ERα, ERβ and AR expression were presented in Supplementary Table 1. The correlation coefficients of ERα and ERβ, ERα and AR, and ERβ and AR expression were 0.275 (p = 0.001), 0.287 (p = 0.001), 0.388 (p < 0.001), respectively.

**Table 1:** Association of expression of ERα, ERβ, and AR with clinicopathological characteristics in gastric cancer [n (%)]

Variable	Total patients (n=155)	Evaluable patients					
		ERα (n=150)*		ERβ (n=153)*		AR (n=139)*	
		Positive No.(%)	*p*	Positive No.(%)	*p*	Positive No.(%)	*p*
Sex							
Female	38	2(5.4)	1.000	35(92.1)	0.710	13(39.4)	0.685
Male	117	7(6.3)		108(93.9)		46(43.4)	
Age, years							
≤58 (median age)	80	3(3.9)	0.318	72(92.3)	0.746	23(32.4)	**0.017**
>58 (median age)	75	6(8.2)		71(94.7)		36(52.9)	
Adjuvant chemotherapy							
Yes	129	6(4.8)	0.357	120(94.5)	0.375	50(42.7)	1.000
No	26	3(12.0)		23(88.5)		9(40.9)	
Tumor Grade							
Undifferentiated	5	1(20)	0.261	4(80.0)	0.067	0(0.0)	**0.009**
Poor	89	4(4.7)		80(90.9)		28(35.0)	
Moderate/well	61	4(6.7)		59(98.3)		31(56.4)	
Vascular invasion							
Yes	91	4(4.5)	0.489	86(96.6)	0.095	35(43.8)	0.717
No	64	5(8.1)		57(91.9)		24(40.7)	
Nerve invasion							
Yes	91	4 (4.5)	0.489	83(93.3)	1.000	29(37.2)	0.155
No	64	5(8.1)		60(93.8)		30(49.2)	
T classificaion#							
pTis	1	0(0.0)	0.565	1(100.0)		0(0.0)	0.617
pT1	5	0(0.0)		5(100.0)		3(60.0)	
pT2	15	1(6.7)		14(93.3)		6(40.0)	
pT3	6	1(16.7)		6(100.0)		4(66.7)	
pT4	128	7(5.7)		117(92.9)		46(41.7)	
N classificaion#							
pN0	39	1(2.6)	0.837	37(94.9)	0.635	17(48.6)	0.841
pN1	20	1(5.0)		18(90.0)		8(40.0)	
pN2	36	3(8.3)		35(97.2)		13(38.2)	
pN3	60	4(7.1)		53(91.4)		21(42.0)	
M classificaion#							
M0	141	7(5.1)	0.199	129 (92.8)	0.600	54(42.5)	1.000
M1	14	2(14.3)		14(100.0)		5(41.7)	
TNM stage#							
I	8	0(0.0)	0.319	7(87.5)	0.348	2(25.0)	0.266
II	39	3(7.7)		38(97.4)		20(55.6)	
III	94	4(4.5)		84(91.4)		32(38.1)	
IV	14	2(14.3)		14(100.0)		5(41.7)	

ERα, estrogen receptor alpha; ERβ, estrogen receptor beta; AR, androgen receptor. #The 7^th^ TNM Classification of Malignant Tumors proposed by the AJCC/UICC. * The number of patients differed between ERα, ERβ and AR since some tissues shed off from the microarray.

### Association of ERα, ERβ and AR expression with clinicopathological characteristics

Clinicopathological characteristics including patient sex, age, whether received adjuvant chemotherapy, tumor grade, vascular and nerve invasion, TNM classification and stage of the patients enrolled in this study were listed in Table 1. Patients with resectable lesion received D2 resection. Among 129 patients received adjuvant chemotherapy, most received standard chemotherapy according to the NCCN guidelines of the year they undergone treatment (2007 to 2010), including XELOX regimen (capecitabine plus oxaliplatin), fluorouracil monotherapy, FOLFOX regimen (oxaliplatin plus fluorouracil), EOF regimen (epirubicin, oxaliplatin plus fluorouracil), and ECF regimen (epirubicin, cisplatin plus fluorouracil). As shown in Table 1, there was no significant association of ERα or ERβ expression with any of the clinicopathological characteristics. However, higher AR positive rate was observed in patients older than 58 years old (median age of the patients) compared to patients younger than 58 years old (*p*=0.017). Moreover, higher AR expression was observed in patients with better differentiated tumors (*p*=0.009).

### Positive expression of ERα was associated with unfavorable outcome in GC patients

**Figure 2:**
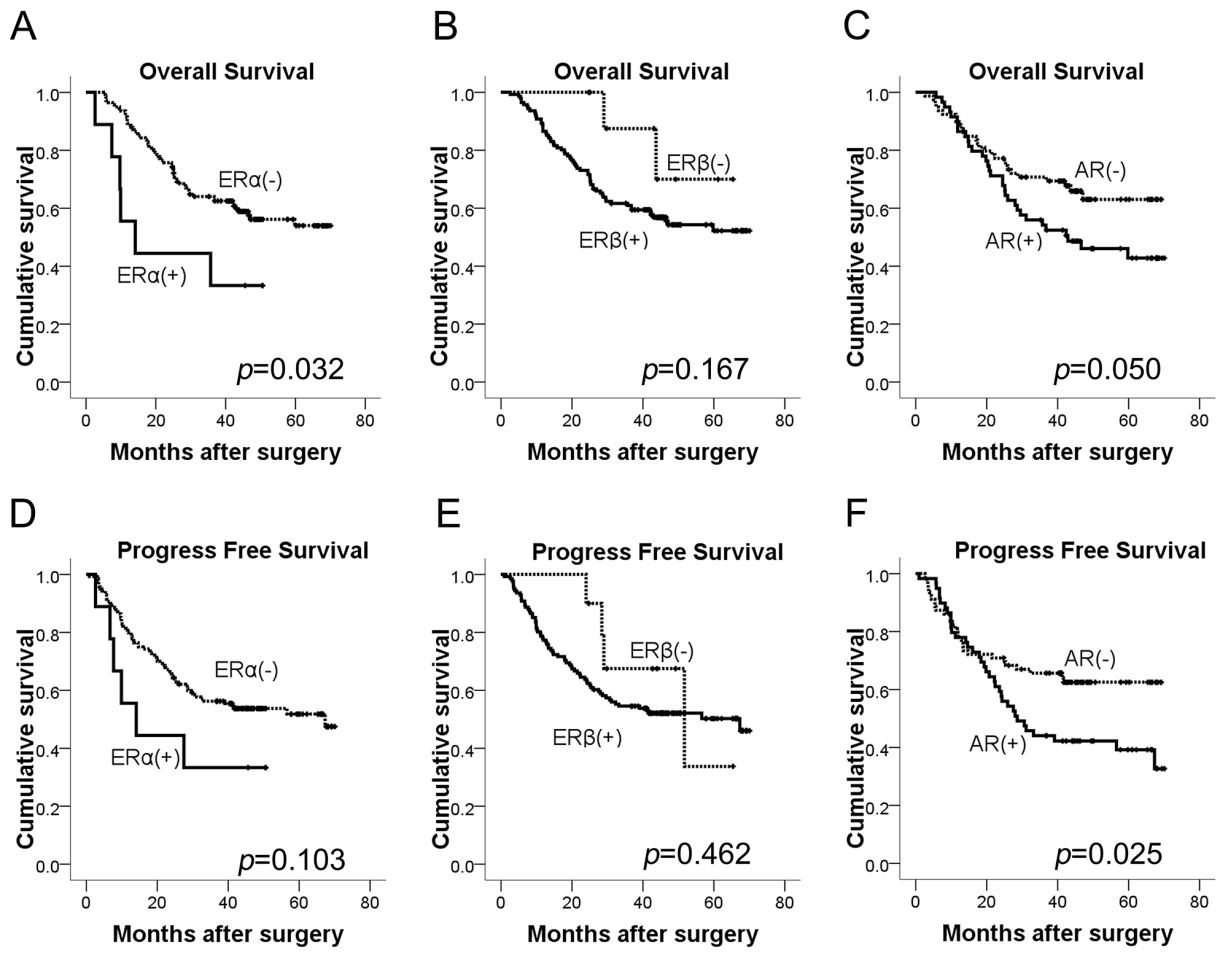
Kaplan-Meier curves of OS and PFS for GC patients according to **(A, D)** ERα, **(B, E)** ERβ, **(C, F)** AR expression (log-rank test). OS, overall survival; PFS, progress free survival.

The median follow-up time for the patients was 48.5 months (range 24.7-70.1 months), and nine patients who failed to contact through telephone or email were lost to follow up. Kaplan-Meier survival curves for ERα, ERβ and AR expression were shown in Figure 2. Three-year overall survival rate for ERα-positive patients was 44.4% (4/9) compared to 59.3% (83/140) for ERα-negative patients (*p*=0.032; Figure 2A), while 3-year progress free survival rate for ERα-positive patients was 44.4% (4/9) compared to 53.6% (75/140) for ERα-negative patients (*p*=0.103; Figure 2D). However, ERβ expression had no significant association with overall survival (OS) (*p*=0.167; Figure 2B) or progress free survival (PFS) (*p*=0.462; Figure 2E) of GC patients. Besides, for patients with positive AR expression, the 3-year overall survival rate was 55.2% (32/58) compared to 63.8% (51/80) for AR negative patients (*p*=0.05; Figure 2C), and 3-year progress free survival rate for AR positive patients was 46.6% (27/58) compared to 60.0% (48/80) for AR negative patients (*p*=0.025; Figure 2F).

Moreover, univariate analysis of OS demonstrated in Table 2 showed the significant prognostic factors for OS included ERα, N classification, M classification and TNM stage; AR was borderline significant associated with OS (p=0.052). ERα, N classification and M classification were retained in multivariate analysis, indicating that in addition to N classification and M classification, ERα was also an independent unfavorable factor for OS of GC patients. Table 3 demonstrated the findings of univariate and multivariate analysis of PFS. The significant prognostic factors for PFS in the univariate analysis included AR, N classification, M classification and TNM stage. However, multivariate analysis showed that AR was only borderline significant after adjustment with other variables (p=0.075), and N classification and M classification turned out to be independent unfavorable factors for PFS of GC patients.

**Table 2:** Univariate and multivariate analysis of OS by Cox model in gastric cancer

Variable	Univariate Cox			Multivariate Cox		
	HR	95%CI	*p*	HR	95%CI	*p*
Sex						
Male	1.000					
Female	1.127	0.635-2.002	0.683			
Age, years						
≤ 58 (median age)	1.000			1.000		
>58 (median age)	1.165	0.722-1.882	0.531	1.367	0.826-2.261	0.224
Adjuvant therapy						
No	1.000			1.000		
Yes	1.136	0.580-2.226	0.709	0.732	0.347-1.544	0.413
T #						
pTis, pT1	1.000			1.000		
pT2	0.977	0.189-5.036	0.977	1.122	0.201-6.277	0.895
pT3	2.881	0.527-15.754	0.222	1.446	0.233-8.990	0.692
pT4	1.380	0.336-5.658	0.655	1.188	0.242-5.832	0.832
N classification #						
pN0	1.000			1.000		
pN1	0.814	0.287-2.313	0.700	0.610	0.210-1.777	0.365
pN2	1.004	0.443-2.276	0.993	0.673	0.280-1.617	0.376
pN3	2.986	1.554-5.736	**0.001**	2.937	1.487-5.799	**0.002**
M classification #						
M0	1.000			1.000		
M1	5.807	3.070-10.986	**<0.001**	7.906	3.978-15.711	**<0.001**
TNM stage						
I	1.000			1.000		
II	4.250	0.563-32.069	0.161	4.100	0.563-32.069	0.174
III	4.114	0.565-29.986	0.163	2.390	0.565-29.986	0.399
IV	22.879	2.956-177.050	**0.003**	-*	-*	-*
ERα						
Negative	1.000			1.000		
Positive	2.436	1.050-5.651	**0.038**	3.639	1.432-9.246	**0.007**
ERβ						
Negative	1.000					
Positive	2.634	0.645-10.762	0.177			
AR						
Negative	1.000					
Positive	1.661	0.995-2.774	0.052			

Abbreviations: HR, hazard ratio; CI, confidence interval; ERα, estrogen receptor alpha; ERβ, estrogen receptor beta; AR, androgen receptor; OS, overall survival. # The 7^th^ TNM Classification of Malignant Tumors proposed by the AJCC/UICC. *TNM stage IV is not calculated since it is linearly correlated with M classification.

**Table 3:** Univariate and multivariate analysis of PFS by Cox model in gastric cancer

Variable	Univariate Cox			Multivariate Cox		
	HR	95%CI	*p*	HR	95%CI	*p*
Sex						
Male	1.000					
Female	1.064	0.632-1.790	0.861			
Age, years						
≤58 (median age)	1.000			1.000		
>58 (median age)	1.224	0.777-1.928	0.384	1.209	0.704-2.077	0.492
Adjuvant therapy						
No	1.000			1.000		
Yes	1.203	0.634-2.281	0.572	0.626	0.291-1.347	0.231
T #						
pTis, pT1	1.000			1.000		
pT2	0.509	0.114-2.273	0.509	0.604	0.125-2.912	0.530
pT3	1.958	0.437-8.776	1.958	0.972	0.164-5.743	0.973
pT4	1.033	0.324-3.291	1.033	1.242	0.296-5.218	0.767
N classification #						
pN0	1.000			1.000		
pN1	0.822	0.316-2.141	0.689	0.862	0.311-2.392	0.776
pN2	0.861	0.391-1.899	0.711	0.681	0.277-1.675	0.402
pN3	2.885	1.573-5.291	**0.001**	3.083	1.565-6.074	**0.001**
M classification #						
M0	1.000			1.000		
M1	6.979	3.724-13.079	**<0.001**	11.907	5.545-25.566	**<0.001**
TNM stage	2.106	1.358-3.267				
I	1.000			1.000		
II	2.106	0.483-9.175	0.321	2.124	0.482-9.366	0.320
III	2.350	0.569-9.698	0.237	1.202	0.278-5.198	0.806
IV	15.320	3.404-68.947	**<0.001**	-*	-*	-*
ERα						
Negative	1.000					
Positive	1.979	0.856-4.573	0.110			
ERβ						
Negative	1.000					
Positive	1.474	0.538-4.042	0.451			
AR						
Negative	1.000			1.000		
Positive	1.739	1.065-2.840	**0.027**	1.573	0.955-2.592	0.075

Abbreviations: HR, hazard ratio; CI, confidence interval; ERα, estrogen receptor alpha; ERβ, estrogen receptor beta; AR, androgen receptor; PFS, progress free survival. # The 7^th^ TNM Classification of Malignant Tumors proposed by the AJCC/UICC. *TNM stage IV is not calculated since it is linearly correlated with M classification.

### Downregulation of ERα suppressed the proliferation, migration and invasion of GC cell lines *in vitro* possibly through regulating the expression of p53, p21, p27, cyclin D1 and E-cadherin

**Figure 3:**
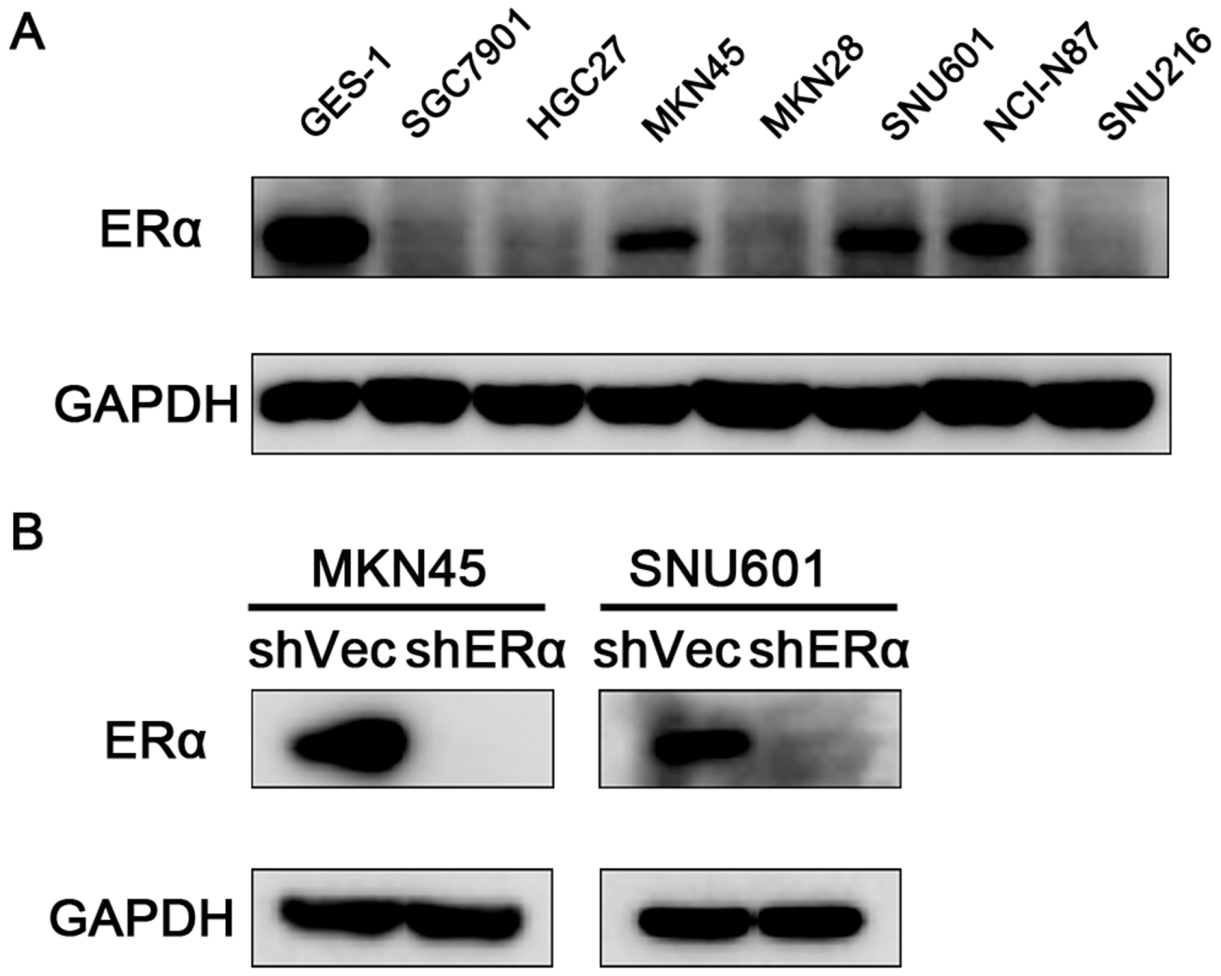
Expression of ERα in GC cells **(A)** Endogenous expression of ERα in the normal gastric mucosal epithelial cell (GES-1) and GC cell lines. **(B)** ERα expression was successfully silenced in MKN45 and SNU601 cells compared with corresponding vector cells.

**Figure 4:**
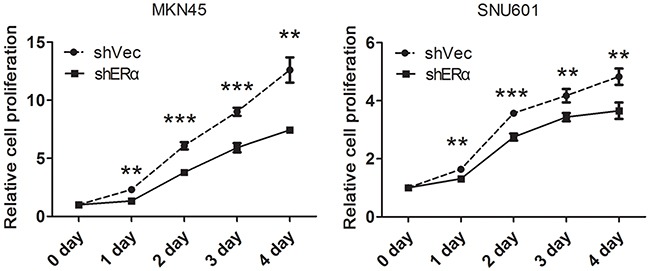
Downregulation of ERα inhibited the proliferation of GC cells *in vitro* The proliferation of GC cells was determined by Cell Counting Kit 8 (CCK8), the proliferation rate of the ERα-silenced MKN45 and SNU601 cells was significantly suppressed compared with corresponding vector cells.

**Figure 5:**
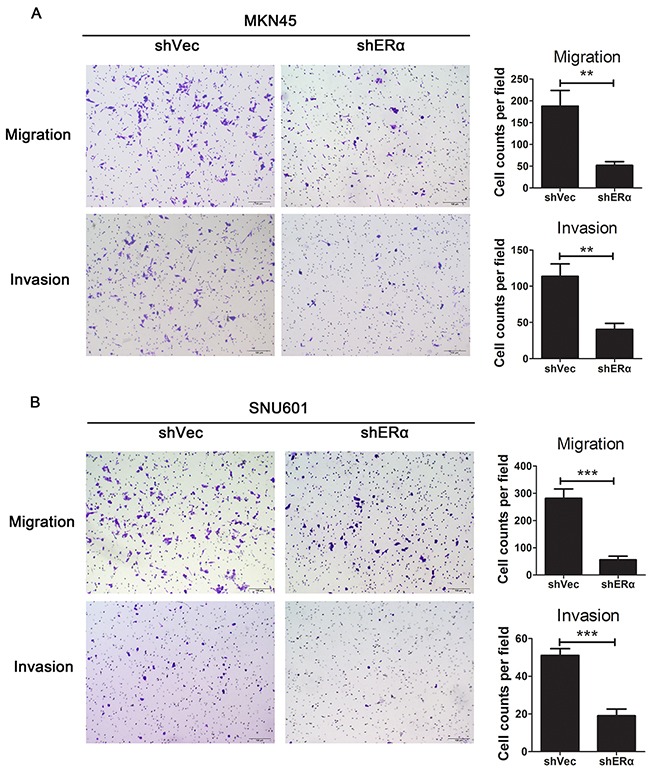
Downregulation of ERα suppressed the migration and invasion of GC cells *in vitro* **(A)** Representative images for ERα stably downregulated or vector transfected MKN45 cells in migration and invasion assays. Numbers for cell counts per field were shown in bar graphs. **(B)** Representative images for ERα stably downregulated or vector transfected SNU601 cells in migration and invasion assays. Numbers for cell counts per field were shown in bar graphs. *p< 0.05; ** *p*< 0.01; *** *p*< 0.001. Results were demonstrated as mean ± standard deviation from three independent experiments.

We next investigated the effects of ERα on the malignant behavior of GC cells. We examined the expression of ERα in GC cell lines, and found that ERα was expressed in low levels in GC cells compared with the normal gastric epithelial cell line (GES-1), which was in accordance with the low positive rate of ERα in GC tissues examined by immunohistochemistry. MKN45 and SNU601 cells with ERα silenced were established by lentivirus infection, as the two cell lines showed relative high basal levels of ERα expression (Figure 3A). Downregulation of ERα was confirmed by western blot (Figure 3B). Knockdown of ERα significantly suppressed the proliferation of MKN45 and SNU601 cells compared with vector cells (Figure 4). Furthermore, ERα silenced MKN45 and SNU601 cells showed significantly decreased migration and invasion in comparison with vector cells (Figure 5).

**Figure 6:**
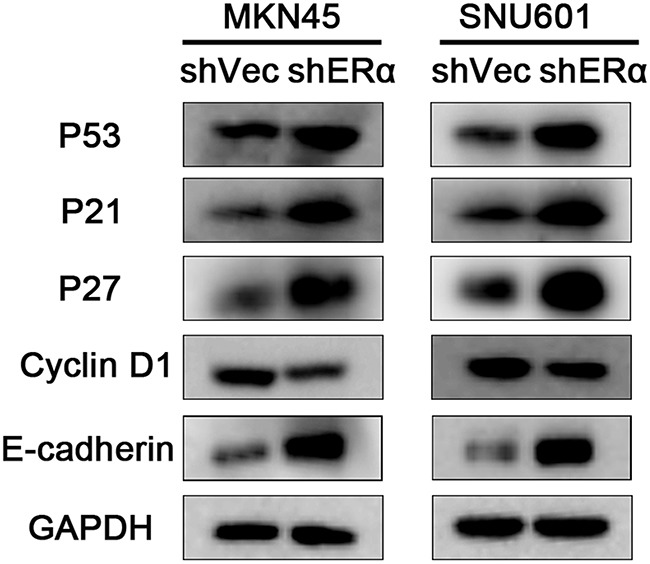
Downregulation of ERα suppressed the proliferation, migration and invasion of GC cells via regulating the expression of p53, p21, p27, cyclin D1 and E-cadherin The expression of proliferation associated molecules including p53, p21, p27, and the EMT associated molecules E-cadherin were increased, while cyclin D1 was reduced in ERα stably downregulated MKN45 and SNU601 cells compared with corresponding vector cells.

To further explore the mechanisms in the decreased proliferation, migration and invasion by ERα knockdown, proliferation associated molecules (p53, p21, p27, cyclin D1) as well as the important molecular in adherens junctions of epithelial cells (E-cadherin) were examined by western blot (Figure 6). The expression of p53, p21, p27 were increased in ERα silenced MKN45 and SNU601 cells, as opposed to cyclin D1, which could explain the suppressed proliferation of ERα knockdown GC cells. In addition, increased expression of E-cadherin was observed in ERα silenced MKN45 and SNU601 cells, which was in accordance with the inhibited migration and invasion of ERα silenced MKN45 and SNU601 cells. These results suggested that downregulation of ERα suppressed the proliferation, migratory and invasive abilities of GC cells probably by enhancing the protein levels of p53, p21, p27 and E-cadherin and inhibiting cyclin D1 expression.

Although the present study found that AR was only borderline significantly associated with poor PFS, considering studies on the expression and function of AR in GC was insufficient, we also investigated the effects of AR on the malignant behavior of GC cells. AR was silenced in MGC803 and SGC7901 cells which showed relative high basal levels of AR expression by lentivirus infection (Supplementary Figure 1). No significant difference was detected in the proliferation of AR silenced cells compared to vector cells (Supplementary Figure 2). However, downregulation of AR significantly suppressed the migration and invasion of MGC803 and SGC7901 cells in comparison with vector cells (Supplementary Figure 3). The expression of epithelial-mesenchymal transition (EMT) associated molecules in AR silenced and corresponding vector cells were examined by western blot (Supplementary Figure 4). The reduced expression of β-catenin, Alpha smooth muscle actin (α-SMA), snail, and slug in AR knockdown MGC803 and SGC7901 cells were in accordance with the suppressed migration and invasion of the two GC cell lines. However, E-cadherin expression was not altered in both AR downregulated cells, perhaps due to the low basal expression of E-cadherin in the two cell lines. These results indicated that downregulation of AR suppressed migratory and invasive abilities of GC cells possibly by inhibiting the EMT pathway.

## DISCUSSION

The present study demonstrated 6% (9/150) of GC tissues expressed ERα, 93.5% (143/153) expressed ERβ, and 42.4% (59/139) were AR positive. The correlation coefficients among ERα, ERβ and AR were too small (r < 0.4), indicating that the correlation was too weak to have any clinical significance in GC. The positive expression of ERα was an independent prognostic factor for OS, and AR was borderline associated with PFS for GC patients after adjustment with other variables. In addition, downregulation of ERα might suppress the proliferation, migration and invasion *in vitro* via regulating the expression of p53, p21, p27, cyclin D1 and E-cadherin. And AR suppressed the migration and invasion of GC cells *in vitro* possibly through inhibiting EMT process.

Treatment of ER positive breast cancer patients with ER antagonist has achieved great success [21]. However, whether GC can benefit from hormonal therapy remains controversial. Earlier in the 1980s, several Japanese studies found that tamoxifen administration could prolong the survival of GC patients [22–24], while an UK study demonstrated that GC patients could not benefit from tamoxifen [25]. Besides, a Korea study published in 2014 indicated that ERα positive GC patients had shorter PFS; Estradiol promoted the proliferation of ERα positive GC cells without affecting ERα negative GC cells, while fulvestrant (an selective ER degrader) abrogated the enhancement of the proliferation of ERα positive GC cells caused by estradiol [17]. It seemed that studies done in different races drew different conclusions. Besides, *ERα* expression patterns varied in different GC patients, for example, the data from TCGA database (The Cancer Genome Atlas, https://tcga-data.nci.nih.gov/docs/publications/tcga/) showed that among 205 stomach adenocarcinoma samples, 5 samples were detected with *ERα* amplification, 1 with deep *ERα* deletion, and 6 with *ERα* mRNA upregulation. In light of this, high-quality clinical trials that examine the effect of anti-ER therapy on the treatment of ER positive GC patients are needed, and anti-ER therapy might be only effective on ER positive individuals.

Limited researches have explored the effect of ERα and the underlying mechanisms in GC. Several previous experimental studies has demonstrated that ERα could promote the proliferation of GC cells possibly by interacting with hedgehog pathway [26], c-Src pathway [27], or cyclin D1 [28], and downregulation of ERα could increase the expression of E-cadherin [17]. Besides, ERα was reported to bind p53 and inhibited the p53-mediated transcriptional activities in breast cancer [29, 30]. On basis of the previous reports and the biological function of ERα revealed by the present study, we examined the expression of several crucial proliferation associated proteins and the important EMT associated protein (E-cadherin), and found enhanced p53, p21, p27 and E-cadherin expression and decreased cyclin D1 expression in ERα silenced GC cells. P53, a typical tumor suppressor protein, is capable of modulating the transcription of various target genes, such as *p21* [29]. P21 and p27 are important cell cycle inhibitors which can prevent the activation of cyclin-dependent kinases including cyclin D/CDK complex [31]. E-cadherin is a crucial protein in maintaining the cell-cell adhesion, loss of which can initiate EMT process [32]. Taken together, knockdown of ERα could suppress the proliferation, migration and invasion of GC cells possibly via modulating the expression of p53, p21, p27, cyclin D1 and E-cadherin.

Since the prognostic role of AR in GC and the potential mechanisms are insufficient, even though the association of AR with PFS only reached borderline significance in the present study, we performed *in vitro* experiment, and revealed that downregulation of AR suppressed the migratory and invasive ability of GC cells, possibly via inhibition of EMT associated molecules including α-SMA, β-catenin, snail, and slug. In fact, Kominea et al firstly reported that AR was an unfavorable prognostic factor for the OS of GC patients [19]. Later Zhang et al examined the expression of AR with a relatively small sample size and showed that AR expression was associated with more lymph node metastasis and later TNM stage, and demonstrated that AR could promote GC metastasis by upregulating MMP9 [20]. Taken together with findings of those two studies and our study, positive AR expression might be an unfavorable factor for the survival of GC patients.

In summary, the present study showed that positive expression of ERα was significantly associated and positive expression of AR had a tendency to associate with poor prognosis of Chinese GC patients. In addition, knockdown of ERα suppressed the proliferation, migration and invasion of GC cells probably via modulating the expression of p53, p21, p27, cyclin D1 and E-cadherin. And downregulation of AR suppressed the migration and invasion of GC cells possibly via inhibiting EMT process. The results suggested that ERα and AR might serve as prognostic biomarkers and therapeutic targets to improve the survival of Chinese GC patients. In the next period of time, precise approaches are needed to identify the expression patterns of ERα and AR in GC patients, and clinical trials involving highly selected ERα and AR positive patients are required to illustrate the potential of anti-ERα and anti-AR therapy in GC.

## MATERIALS AND METHODS

### Patients and clinicopathological data

Formalin-fixed and paraffin-embedded tumor tissues were collected from 155 patients with gastric carcinoma who underwent surgical resection at the Department of Gastric Cancer and Soft Tissue Sarcomas, Shanghai Cancer Center of Fudan University, Shanghai, China, from Oct 2007 to Jan 2010. The samples were used for tissue microarray construction and immunohistochemistry. The patients were followed up every 4 months until death or the end of the study (September 2nd, 2013), except those lost to follow up. Overall survival (OS) was defined as the interval between the date of surgery and the date of death or last follow-up visit. Progress free survival (PFS) was defined as the time from the date of surgery to the time of disease progression, death, or last follow-up visit if the disease did still not progress.

Informed consents were obtained from all patients, and the research was approved by the Clinical Research Ethics Committee of Fudan University Shanghai Cancer Center, and complied with the principles of the Helsinki Accord.

### Immunohistochemistry

ERα, ERβ and AR expression was detected by immunohistochemistry using UltraSensitive™ SP kit (#9710, Maixin, Fuzhou, China) according to the manufacturer's instructions. In brief, the sections were deparaffinized, rehydrated and subjected to antigen retrieval (citrate buffer, pH=6.0). The sections were then incubated overnight at 4°C with the primary mouse monoclonal antibodies to ERα (clone 33, ab2746, Abcam; 1:50), ERβ (clone 14C8, ab288, Abcam; 1:100) and AR (clone AR 441, ab9474, Abcam; 1:200), respectively. The sections were subsequently washed and incubated with a secondary antibody. Reaction products were visualized with 3, 3’diaminobenzidine tetrahydrochloride and counterstained with hematoxylin and eosin.

### Staining evaluation

Cases displaying brown cytoplasmic and/or nuclear stainings were regarded as positive. The staining intensity was graded by the Allred score system [33]. A score≥3 was considered as positive and a score less than 3 was designated as negative. The immunoreactivity was viewed by two pathologists independently.

### Cell culture

Nine human GC cell lines (MGC803, AGS, SGC7901, HGC27, MKN45, MKN28, SNU601, NCI-N87, SNU216), one normal gastric epithelial cell line (GES-1) and HEK293T cell line were obtained from the Cell Resource Center, Shanghai Institute of Biochemistry and Cell Bank at the Chinese Academy of Sciences. All cell lines were routinely authenticated by DNA-fingerprinting and isoenzyme analysis and free of contamination by mycoplasma. GC cell lines were cultured in RPMI 1640, MEM, or DMEM containing 10% fetal bovine serum at 37 °C under humidified atmosphere with 5% CO2.

### Lentivirus production and transduction

Lentiviral vector expressing shRNA targeting ERα was purchased from Obio Tech (Shanghai, China), and lentiviral vector expressing shRNA targeting AR was purchased from Genechem (Shanghai, China). Those shRNAs were transfected into HEK293T cells using FuGene^®^ HD Transfection Reagent (Promega) to generate lentivirus. MKN45, SNU601, MGC803 and SGC7901 cells were infected with the recombinant lentivirus with 10μg/mL Polybrene^®^ (Sigma-Aldrich, St Louis, MO, USA).

### Cell proliferation assays

MKN45 and SNU601 cells were plated at a density of 2000 cells per well in 96-well plates. MGC803 and SGC7901 cells were plated at a density of 1000 cells per well in 96-well plates. Cell proliferation was determined with Cell Counting Kit 8 (CCK8, Dojindo, Kumamoto, Japan) for 5 days.

### Cell migration and invasion assays

Migration and invasion assays *in vitro* were carried out in chambers of 8-μm transwell inserts (BD Falcon™; Becton Dickinson, Franklin Lakes, NJ, USA) in the presence or absence of Matrigel (BD Falcon™). Eighty thousand MKN45 and SNU601 cells, 40,000 MGC803 cells and 60,000 SGC7901 cells were seeded in the top chamber in serum-free medium, while medium containing 20% serum was in the lower chamber as the attractant. After 24-48 hours, migrated cells were fixed with 4% paraformaldehydeand then stained with 0.1% crystal violet. The number of migrated cells was counted using an IX71 inverted microscope (Olympus Corp, Tokyo, Japan).

### Western blot

Cells were lysed and the protein concentration was measured using the bicinchoninic acid protein assay kit (Biyotime, Shanghai, China). The lysates were subjected to SDS-PAGE gel and transferred to a PVDF membrane (Millipore, Billerica, USA). The membrane were blocked with 5% non-fat milk and incubated with indicated primary antibodies, and then probed with the horseradish peroxidase–conjugated secondary antibody. The primary antibodies against ERα was purchased from Abcam. The primary antibodies against p53 and p27 were purchased from Santa Cruz Biotechnology. The primary antibodies against p21 were purchased from Abways Technology. The primary antibodies against AR, snail, slug, α-SMA, and β-catenin were purchased from Cell Signaling Technology. The antibody against GAPDH was purchased from Proteintech Group. The blots were visualized with Pierce™ enhanced chemiluminescence reagents (Life Technologies).

### Statistical analysis

The associations of ERα, ERβ and AR expression with clinicopathological characteristics were evaluated by Chi-square test or Fisher's exact test. Survival data were analyzed using Kaplan-Meier method (log-rank test). Cox proportional hazards was used for univariate and multivariate survival analysis, clinical factors considered relevant for the prognosis including age, adjuvant therapy, TNM classification and stage according to the REMARK criteria [34], as well as other significant (p <0.05) factors in the univariate analysis were allowed to enter multivariate analysis, and a reduced model was applied using stepwise backward elimination until only significant (*p*<0.05) variables remained in multivariate survival analysis. The data of functional experiments were expressed as the mean ± standard deviation from three independent experiments, and analyzed using Student's *t*-test. All statistical analyses were performed by the SPSS 19.0 for windows (SPSS Inc, Chicago, IL, USA). A two-tailed *p*< 0.05 was considered statistically significant.

## SUPPLEMENTARY MATERIALS FIGURES AND TABLE



## Abbreviations

ER: estrogen receptor
AR: androgen receptor
GC: gastric cancer
