# Supplementary material for: Expression of estrogen receptors and androgen receptor and their clinical significance in gastric cancer
Source: Oncotarget. 2017 Mar 28;8(25):40765–77. doi: 10.18632/oncotarget.16582 (PMC5522298; doi:10.18632/oncotarget.16582)
Supplement: Supplementary file 1 [file oncotarget-08-40765-s001.pdf]

Expression of estrogen receptors and androgen receptor and their clinical significance in gastric cancer

SUPPLEMENTARY FIGURES AND TABLE

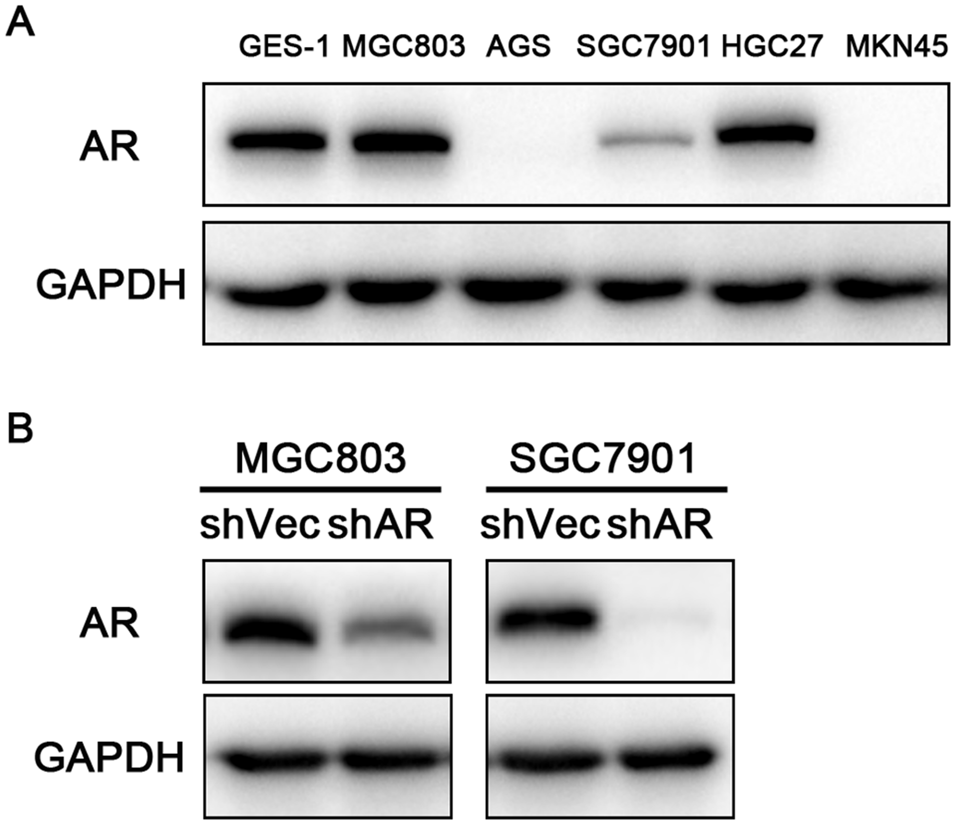

Supplementary Figure 1: Expression of AR in GC cells. (A) Endogenous expression of AR in the normal gastric mucosal epithelial cell and GC cell lines. **(B)** AR expression was successfully downregulated in MGC 803 and SGC7901 cells compared with corresponding vector cells.

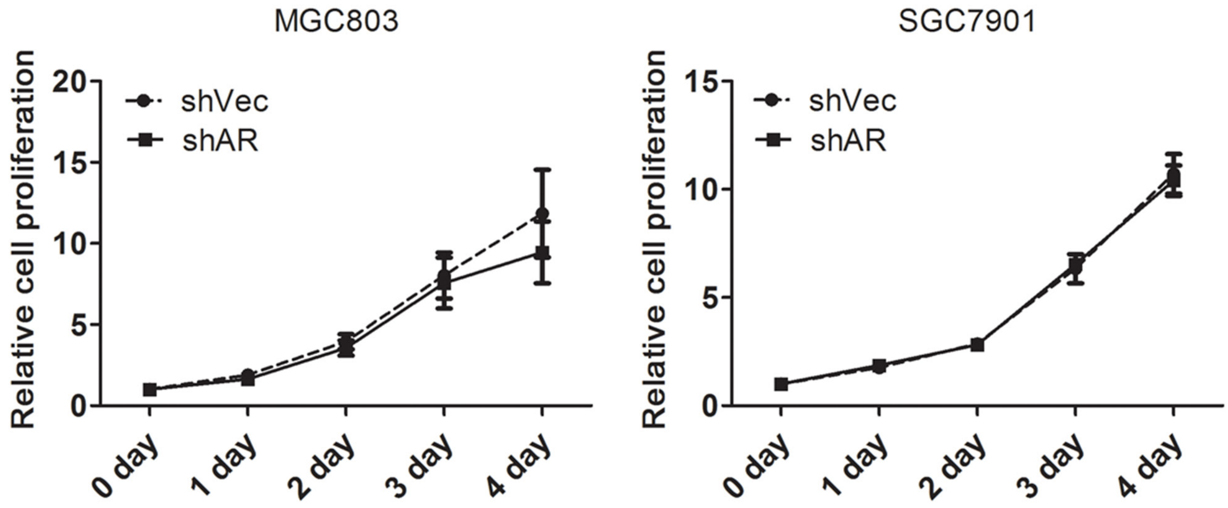

Supplementary Figure 2: Downregulation of AR did not influence the proliferation of GC cells *in vitro*. The proliferation of GC cells was determined by Cell Counting Kit 8 (CCK8), the proliferation rate of the AR downregulated MGC 803 and SGC7901 cells had no difference compared to corresponding vector cells.

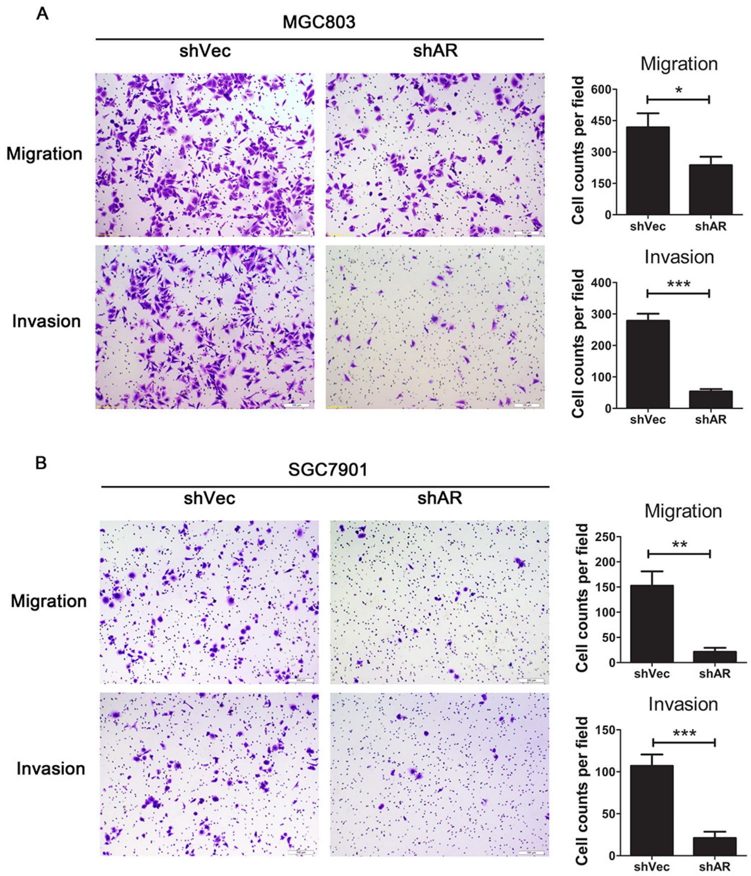

Supplementary Figure 3: Downregulation of AR suppressed the migration and invasion of GC cells *in vitro*. (A) Representative images for AR stably downregulated or vector transfected MGC803 cells in migration and invasion assays. Statistics for cell counts per field were shown in bar graphs. **(B)** Representative images for AR stably downregulated or vector transfected SGC7901 cells in migration and invasion assays. Statistics for cell counts per field were shown in bar graphs. *$p < 0.05$; ** $p < 0.01$; *** $p < 0.001$. Results were the the mean ± standard deviation from three independent experiments.

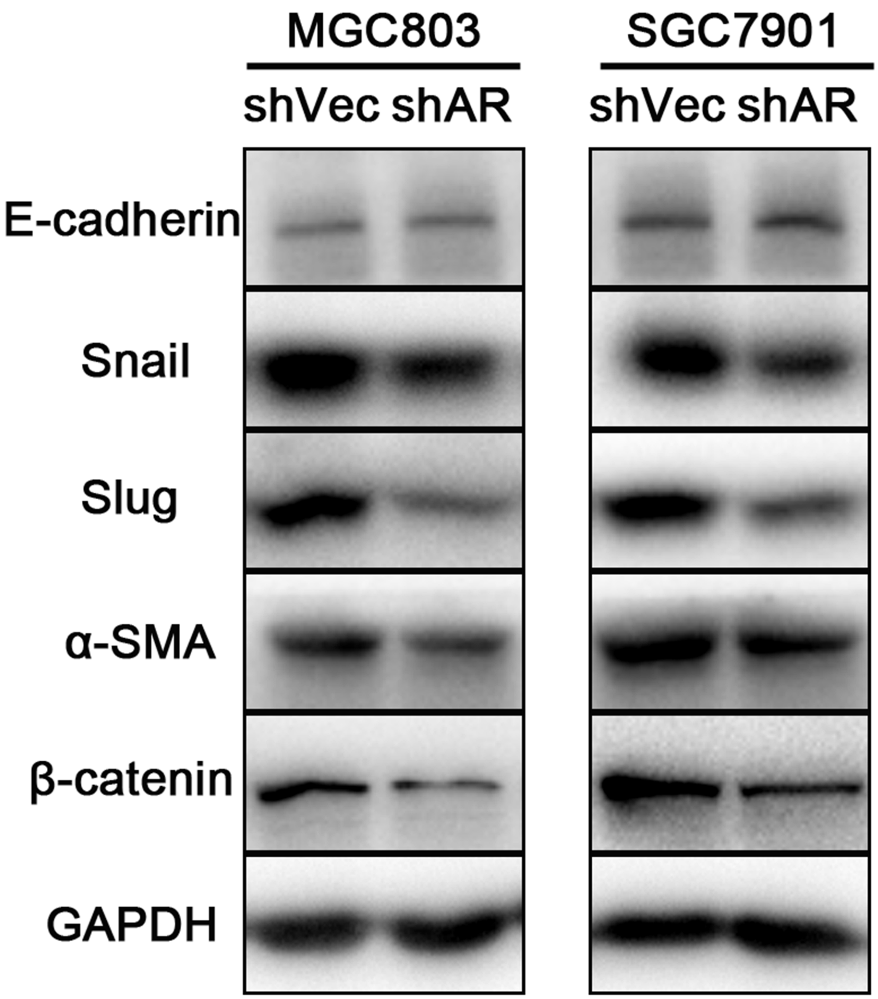

Supplementary Figure 4: Downregulation of AR suppresses the migration and invasion of GC cells via inhibiting EMT. The expression of EMT associated molecules including α-SMA, snail, slug and β-catenin were reduced in AR stably downregulated MGC 803 and SGC7901 cells compared with corresponding vector cells.

Supplementary Table 1: Correlations among expression of ERα, ERβ, AR in gastric cancer

**Correlation**	***r***	***p***
ERα vs ERβ	0.275	0.001
ERα vs AR	0.287	0.001
ERβ vs AR	0.388	<0.001

ERα, estrogen receptor alpha; ERβ, estrogen receptor beta; AR, androgen receptor. r, Spearman rank correlation coefficients.
